# Pancreaticopleural and pancreaticomediastinal fistula: a retrospective case series on clinical outcomes of endoscopic stent therapy in 48 patients

**DOI:** 10.1007/s00464-026-12590-2

**Published:** 2026-02-23

**Authors:** Mike Bäck, Outi Lindström, Mia Rainio, Tuomas Kaprio, Leena Kylänpää, Marianne Udd

**Affiliations:** 1 Faculty of Medicine, University of Turku, Turku, Finland; 2 Department of Gastrointestinal Surgery, Helsinki University Hospital and University of Helsinki, Helsinki, Finland

**Keywords:** Pancreaticopleural fistula, Pancreaticomediastinal fistula, Pancreatitis, ERCP, Pancreatic duct stenting, Endoscopic therapy

## Abstract

**Background:**

Pancreaticopleural and pancreaticomediastinal fistulas (PPMF), are rare complications of pancreatitis caused by a disrupted pancreatic duct. Their optimal management remains unclear due to limited clinical data. The aim of this study was to evaluate outcomes of endoscopic stent therapy in patients with PPMF secondary to acute or chronic pancreatitis.

**Methods:**

A retrospective case series was performed on 59 patients with PPMF treated at Helsinki University Hospital between 2011 and 2024. Final analysis included 48 patients. Diagnosis was based on amylase-rich pleural effusion (>  200 U/L) and/or a fistula tract presented through imaging. The primary treatment method was endoscopic retrograde cholangiopancreatography (ERCP) with transpapillary pancreatic duct (PD) stenting. Treatment success was defined as resolution of pleural fluid and a radiologically identified closure of the fistula.

**Results:**

The main etiology was alcohol-induced pancreatitis (94%). Fistulas communicated with the left pleural cavity in 54%, right in 21%, both in 19%, and mediastinum in 10% of cases. Endoscopic therapy was successful in 90% of patients, with a median of three (range 1–8; IQR 2) ERCPs per patient. Following stent placement, fistula closure occurred after a median of 2.3 months (range 1–24; IQR 3). In 22 (73%) cases, the stent was positioned beyond the ductal disruption, resulting in treatment success in all cases. Complications were rare, including three mild-to-moderate post-ERCP pancreatitis (1.9%) and a few stent-related events that were treated endoscopically. There were no recurrences. Four patients required surgery due to failed endoscopic therapy. Overall mortality was 27%, unrelated to PPMF.

**Conclusions:**

Endoscopic stent therapy is a highly effective and safe treatment method for PPMF. Stent placement beyond ductal disruption is associated with successful treatment. These findings support endoscopic therapy as the first-line treatment for PPMF secondary to acute and chronic pancreatitis. Future prospective studies with larger cohorts are needed to confirm these results.

Pancreatic fistula is an infrequent but well-known complication linked to disruption of the pancreatic duct (PD). It involves an abnormal passage through which enzyme-rich pancreatic fluid may leak into the peritoneal and thoracic cavities, resulting in an internal pancreatic fistula (IPF), or externally to the skin, forming an external pancreatic fistula (EPF) [1]. Pancreatic fistulas are commonly associated with acute and chronic pancreatitis or pancreatic trauma [2]. Apart from post-operative pancreatic fistulas, the classification and consensus on the management of IPF and EPF are lacking due to their rarity [3].

Limited success of traditional treatments for IPF, such as conservative therapy and surgery, has shifted the focus to endoscopic transpapillary pancreatic stent therapy [2]. It is not clear, how stent types and their positioning relative to the fistula affect clinical outcomes [4]. Further categorization of IPF includes the pancreaticopleural fistula (PPF), which is exceedingly uncommon, estimated 0.4% of patients presenting with pancreatitis [5], and pancreaticomediastinal fistula (PMF), which has few published cases [6, 7]. Previous clinical studies on PPF have reported a small cohort of patients; seven [5], four [8], three cases [9], the largest being 22 cases [10] or a study with 27 cases that also included patients with pancreatic ascites [11]. The aim of this study was to evaluate outcomes of endoscopic stent therapy in a larger cohort of patients with pancreaticopleural or -mediastinal fistulas (PPMF). Herein, we report our treatment results with endoscopic stent therapy in 48 patients with PPMF due to acute or chronic pancreatitis.

## Patients and methods

**Fig. 1:**
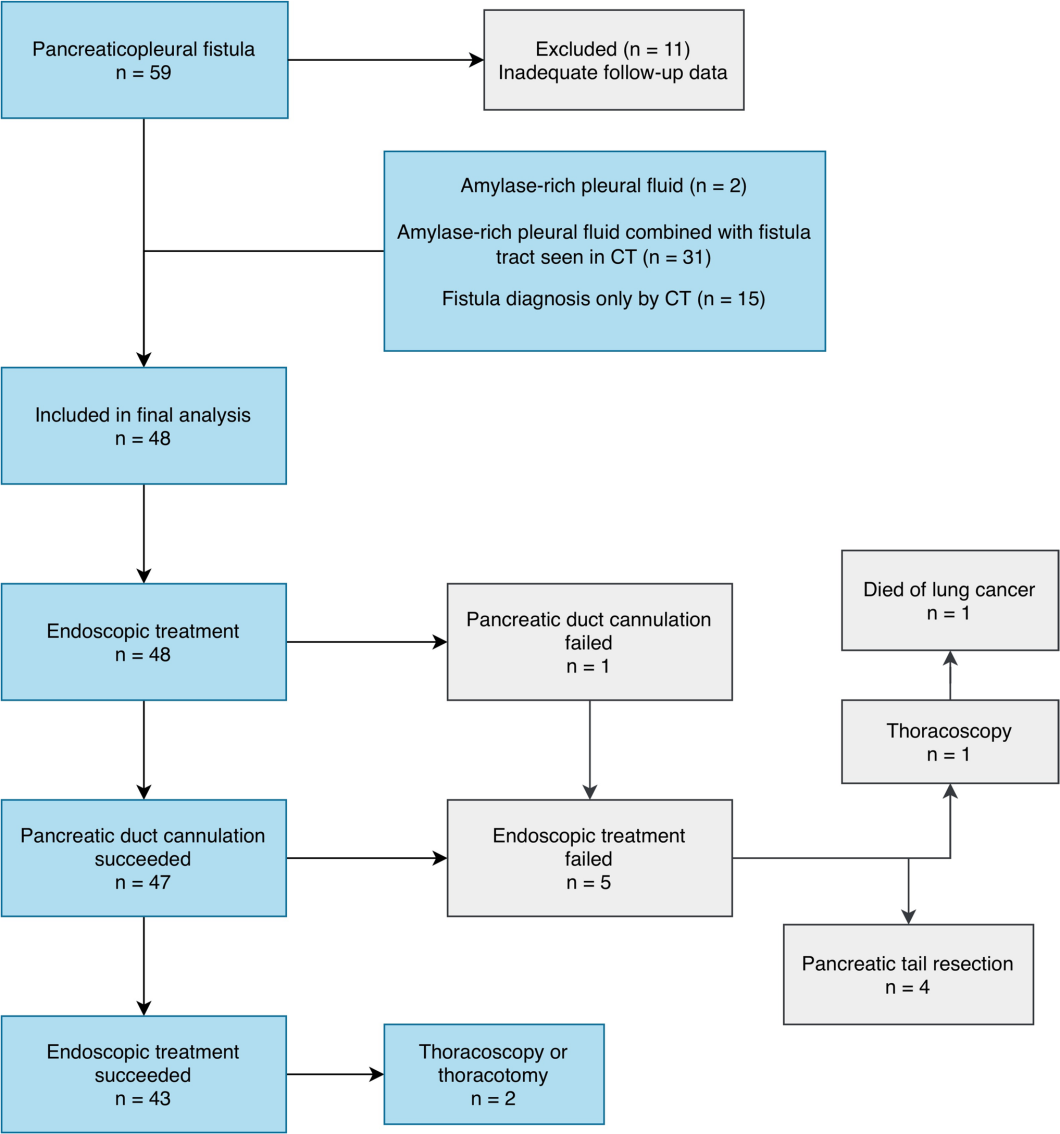
Flowchart of enrolled patients with pancreaticopleural fistula

A retrospective, single-center case series was performed involving 59 patients with PPMF secondary to acute or chronic pancreatitis. Their treatment was initiated endoscopically in Helsinki University Hospital between 2011 and 2024. In Helsinki University Hospital, the standard first-line therapy for pancreatic fistulas has predominantly been endoscopic. Eleven patients were excluded due to inadequate follow-up information, resulting in a final cohort of 48 patients (Fig. 1). The research protocol was approved by the hospital's review board (HUS/176/2025 6.5.2025). Given the retrospective nature of the study, informed consent was not required, and patient or public involvement was not applicable.

**Fig. 2:**
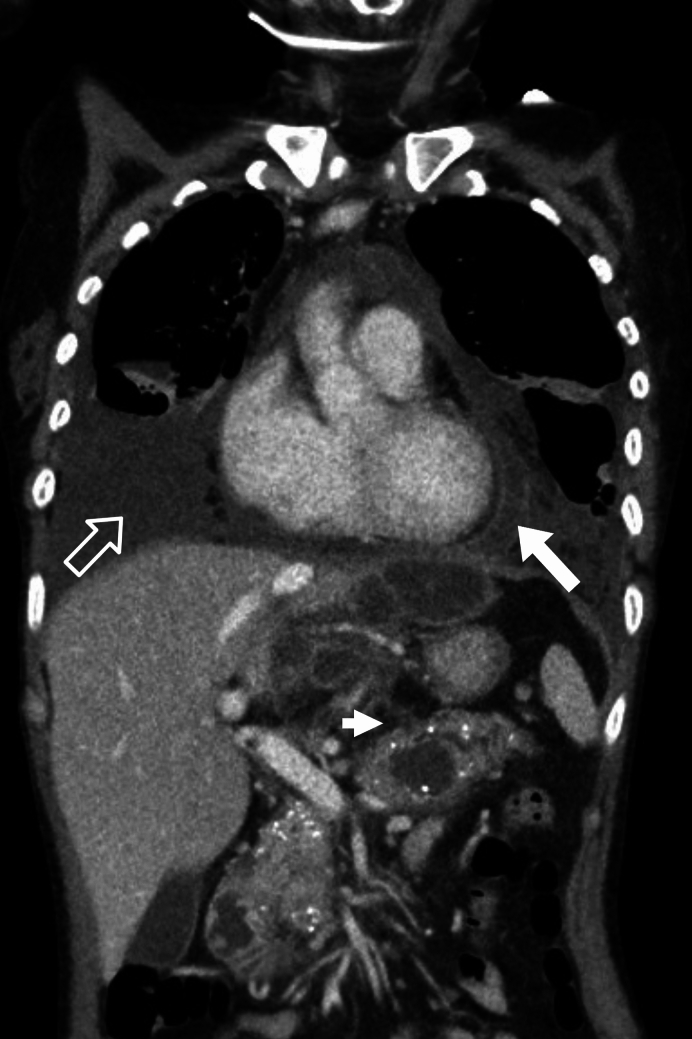
CT demonstrated a fistula tract extending from the body of the pancreas into the right pleural space. Amylase rich fluid in pericardium (bold arrow). Amylase rich fluid in right pleural cavity (open arrow). Location of the fistula (short arrow)

**Fig. 3:**
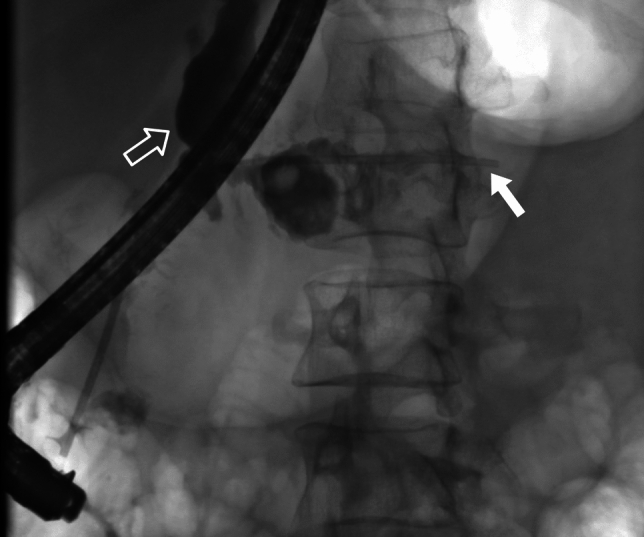
Plastic stent in pancreatic duct placed in ERCP (bold arrow). Pancreatic fistula (open arrow)

All included patients had a PPMF, which was at minimum established through amylase-rich pleural fluid (greater than 200 U/L) or a radiologically identified fistula tract to the pleural cavity and/or to the mediastinum (Figs. 2 and 3). All patients had fluid in the pleural cavity, mediastinum or both.

Chronic pancreatitis was defined as a persistent inflammation of the pancreas leading to either radiologically detectable structural changes, such as calcifications or atrophy, or functional impairment, such as exocrine deficiency. Duct disruption was classified as a partial tear in the PD, whereas a disconnected duct indicated complete interruption of the ductal continuity.

Successful outcome was determined when pleural fluid cleared, and the fistula was no longer visible through imaging. If, after primarily successful treatment, the fistula reappeared causing pleural effusion, this was classified as a recurrence. Follow-up was counted primarily from removal of PD stent. In cases where transpapillary treatment failed and treatment continued transmurally, follow-up was measured from the time of pleural fluid resolution.

All available data, such as information on patients’ characteristics, computed tomography (CT), procedures and endoscopy-related adverse events, was retrospectively collected from patient records. Overall mortality was provided by the Finnish Population Registration Center and Statistics Finland. Plasma amylase was routinely measured 4 h after endoscopic retrograde cholangiopancreatography (ERCP), and on the following morning for patients who stayed overnight. Post-ERCP pancreatitis (PEP) was diagnosed if plasma amylase levels were more than three times higher than the upper limit of reference value at 4 h or 24 h after the procedure, and abdominal pain lead to a prolonged hospital stay. Severity of PEP was categorized according to the Revised Atlanta criteria [12]. Other complications, such as cholangitis, perforation and bleeding, were evaluated according to Cotton’s classification [13].

Data are reported as the number of cases and percentage or as median value, range and interquartile range (IQR). We tested for statistical significance in relation to treatment outcome using Fisher's exact test and considered two-sided *p*-values < 0.05 as significant. Statistical analyses were performed using SPSS version 29 (IBM, Armonk, NY).

### Endoscopic therapy

ERCP with transpapillary access to the PD and subsequent stent placement was the preferred method for endoscopic therapy (ET). Patients were in prone position and anesthesiologist provided conscious sedation. In ERCP, the aim was to identify the fistula tract and place a plastic stent (5Fr-10Fr) beyond the site of ductal disruption. If the first ERCP was unsuccessful and the fistula was still visible in CT, a second attempt was scheduled. Pancreatic sphincterotomy was performed in all cases. When indicated, PD dilatation was performed using a 4–6 mm dilatation balloon.

A follow-up CT was scheduled two months after placement of the first stent. If the pleural effusion had resolved by that time and no fistula was visible on CT, stent removal was planned 8 to 12 months later. Otherwise, PD stent removal was scheduled during stent exchange once the pleural effusion had resolved and the fistula was no longer visible on CT or ERCP. After the fistula was treated, patients with chronic pancreatitis often required additional ERCPs due to PD strictures or stones, mainly for upsizing the stent from 7 to 10Fr, placement of multiple stents or stone removal.

The 5Fr and 7Fr stents were straight, whereas 8.5Fr and 10Fr stents were S-shaped. Occasionally, the fluid collection was in the head of the pancreas, and the guidewire reached the collection rather than the PD behind it. In such cases, transpapillary 7Fr or 10Fr pigtail stents were used first but were replaced with a PD stent once the fluid collection had drained.

If ERCP failed, an endoscopic ultrasound (EUS)-guided transmural route was considered for suitable fluid collections. For transmural drainage, either lumen apposing metal stents or 7Fr double pigtail stents were used.

## Results

Among the patients, 33 (69%) had chronic pancreatitis and 15 (31%) had acute pancreatitis. The etiology of acute or chronic pancreatitis was alcohol in 45 (94%) cases. Of the remaining, one had biliary pancreatitis, one had interferon-induced pancreatitis, and one had idiopathic pancreatitis. Among the patients with alcohol related pancreatitis, 39 (87%) were defined as heavy alcohol users (>  50 g of alcohol per day). Patients’ characteristics are described in Table 1.

**Table 1:** Patients characteristics

	*n* = 48 (%)	*p*-value
Age, years^a^	54 (32–75; 12)	
Sex, male/female	29/19 (60/40)	0.372
BMI kg/m^2 a^	23.1 (15.2–43.9; 6.4)	
Comorbidities		
Cardiovascular	27 (56)	
Pulmonary	13 (27)	
Diabetes	8 (17)	
Other	20 (43)	
CCI^a^	2 (0–8, 2)	
Cigarette smoking	38 (81) *n* = 47	
Etiology of the PPMF		0.307
Acute pancreatitis	15 (31)	
Chronic pancreatitis	33 (69)	
Etiology of acute pancreatitis		
Alcohol	12 (80)	
Biliary	1 (7)	
Idiopathic/Unknown	1 (7)	
Interferon	1 (7)	
Etiology of chronic pancreatitis		
Alcohol	33 (100)	
Alcohol use discontinued after diagnosis	17 (35)	1.000
Symptom		
Shortness of breath	37 (77)	
Abdominal pain	30 (63)	
Chest pain	10 (21)	
Fever	16 (33)	
Pancreatic exocrine insufficiency	4 (8.3)	0.366
CRP (mg/L) in ERCP day^a^	75 (3–344; 93)	
Leucocyte (cells/μL) in ERCP day^a^	9.5 (3.8–23.3; 6.5)	
Symptom duration before endoscopic treatment(days)^a^	14 (1–240; 23) *n* = 47	
Pleurocentesis	27 (56)	
Pleural drainage	25 (52)	
Pleural fluid amylase (U/L)^a^	1706 (6–68,757; 5099) *n* = 38	
CT identified fistula tract	46 (96)	
Pseudocyst / WON	34/2 (71/4)	
Pancreatic calcifications	25 (52)	0.180
PPMF localization		
Pancreatic head	15 (31)	
Pancreatic body	15 (31)	
Pancreatic tail	18 (38)	0.059

*CCI* Charlson Comorbidity Index (age adjusted) [21], *PPMF* pancreaticopleural or pancreacticomediastinal fistula, *ERCP* endoscopic retrograde cholangiopancreatography, *CT* computed tomography, *WON* walled of necrosis,

^a^median (range; IQR)

Reported *p*-values indicate the association of each variable with treatment outcome

CT was performed for all patients and demonstrated the fistula tract in 46 (96%) cases, and three patients also underwent magnetic resonance imaging (MRI). Pleural amylase levels were measured in 38 (79%) cases. During ERCP, the fistula tract was visualized in 38 patients out of 47. The median duration from the onset of an acute pancreatitis episode to the diagnosis of PPMF was six (range 1–240; IQR 10) months. After diagnosis of chronic pancreatitis, the median time for PPMF detection was two (range 0–97; IQR 9) months. Symptoms of PPMF lead to CT and resulted in a diagnosis of chronic pancreatitis in 13 (18%) cases. Typical symptoms were shortness of breath, abdominal pain and chest pain.

Fistula location was the pancreatic head in 15 cases, the body in 15, and the tail in 18. PPMF communicated with the left pleural cavity in 26 (54%) cases and with the right pleural cavity in 10 (21%). Nine (19%) patients had pleural fluid in both sides and in 4 of those instances the fistula communicated also with the mediastinum. Pancreatic fluid perforated to the mediastinum and only to the left pleural cavity in one case. In two cases, the fistula tract went solely to the mediastinum. Three patients had pancreatic ascites as well.

ET was ultimately successful in 43 (90%) patients (Fig. 1, Table 2). Transpapillary access into the PD succeeded in all but one instance. Cannulation of PD succeeded during the first ERCP in 42 cases. Four patients required two ERCP attempts, whereas one patient underwent three ERCP attempts before PD cannulation succeeded. In the one case in which PD cannulation was unsuccessful, two attempts were made. In four cases, stenting was performed through the minor papilla.

**Table 2:** Treatment and outcome

	*n* = 48 (%)	*p*-value
Endoscopic treatment success	43 (90)	
Pancreatic duct cannulation succeeded	47 (98)	
Stent passed beyond the fistula tract	22 (73)	0.267
Symptoms resolved after successful stenting (months)^a^	2 (0.1–14; 1.8)	
Stenting time until fistula resolved (months)^a^	2 (1–24; 2.75)	
Operative treatment		
Distal pancreatic resection	5^b^ (10)	
Laparotomy for abscesses drainage	1 (2.1)	
Transgastric necrosectomy	1 (2.1)	
Thoracotomy	1 (2.1)	
Thoracoscopy	2 (4.2)	
Follow-up after stent removal (months)	54 (1–209; 56)	
Mortality	13 (27)	

*ERCP* Endoscopic retrograde cholangiopancreatography

^a^median (range; IQR)

^b^One because of splenic artery bleeding,

Reported *p*-value indicates the association of each variable with treatment outcome

CT was scheduled approximately two months after the first ERCP and demonstrated a closure of the fistula in 20 (42%) cases. The median time from successful stent placement to PPMF closure was 2.3 months (range 1–24; IQR 3). Among 30 patients with a fistula in the head or body of the pancreas, the stent was successfully positioned past the disrupted area in 22 cases. All of them had a successful treatment outcome. After the stent bypassed the fistula tract, the patients’ symptoms resolved in a median time of 60 (range 3–156; IQR 45) days. Of the remaining eight cases, ET failed in one case. When the fistula was in the tail of the pancreas, ET succeeded in 14 out of 18 patients (77.8%) but when it was in the head or body of the pancreas ET succeeded in 29 out of 30 patients (96.7%) (*p* = 0.059; Table 1).

In the remaining 21 successful ETs, the stent could not be advanced past the site of ductal obstruction. This was primarily due to the fistula being located in the tail of the pancreas in 14 successful endoscopic therapies. In these instances, it was not possible to verify whether the stent had been positioned past the fistula. Remaining seven cases in which the stent was not placed past the injury site included two cases which were treated transmurally. Additionally, in five cases, the stent was placed at the site of ductal obstruction, but not beyond it, yet the fistula resolved. This is presumably because stent placement sufficiently improved ductal flow conditions, allowing pancreatic secretions to drain to duodenum. During each stent exchange, advancement of the stent beyond the site of ductal obstruction was always attempted. If this was not feasible, the stent was positioned as close as possible to the fistula opening.

Patients underwent a median of three (range 1–8; IQR 2) ERCPs. In all 158 ERCPs, there were three PEPs (1.9%), including one moderate and two mild cases. In one patient, during an elective ERCP, it was revealed that distally migrated PD stent had perforated the duodenal wall. Subsequently, the stent was removed, and no additional treatment was needed. In one instance, the PD stent was impacted within the PD, and in another, the stent migrated further into the PD. Both complications were resolved by placing another stent beside the problematic stent, and afterward both stents were successfully removed. PD stenting of one patient was presumed to cause an infection of walled-off necrosis, requiring transgastric necrosectomy.

In some instances, patients with chronic pancreatitis required more procedures after undergoing successful ET, median of three (range 2–8; IQR 3), than patients with acute pancreatitis, median three of (range 2–4; IQR 2). Strictures or stones within the PD in patients with chronic pancreatitis necessitated additional procedures even after resolution of pleural effusion. In acute pancreatitis, one successfully placed stent was usually sufficient.

Overall, four (8%) patients underwent pancreatic surgery after ET failed to achieve resolution. In all of these cases, the fistula was located in the tail of the pancreas, and none of them required thoracic surgery. One patient required surgery as the cannulation of PD was not possible; a pancreatic resection was performed two months after first ERCP, following two unsuccessful cannulation attempts. In the remaining three cases, fistula was not resolved despite successful placement of a 15 cm stent. One of them underwent tail resection soon after stent placement due to severe infection, so the true benefit remained unclear. Only one of those four patients stopped alcohol consumption. Due to empyema, three (6%) patients needed thoracic surgery 4, 5 and 7 days after the first ERCP (Fig. 1, Table 2).

Duct disconnection complicated ET in six cases. In two of these patients the PD stent did not resolve the fistula and transmural drainage with permanent pigtail stents was required. Both had the fistula located in the head of the pancreas and presented with pseudocysts. Endoscopic cystogastrostomy was performed in these patients one week after the first ERCP for one patient, and three months after the first ERCP for the other. In three cases, a pseudocyst was treated transmurally after PD stenting had resulted in resolution of the fistula. The remaining one patient was ET treatment failure but died of lung cancer without undergoing any further procedures.

The median follow-up time was 54 months (range 1–209; IQR 56). There were no fistula recurrences among any of the 48 patients. In one instance the original site of disruption healed, but thereafter the patient continued heavy alcohol consumption. Thus, a new fistula developed in another part of the PD, which was not classified as a recurrence.

There were a considerable number of comorbidities among the patients, especially in those with chronic pancreatitis (Table 1). Mortality was high (27%) but none died due to PPMF. One year mortality from the first ERCP was 4.2%. The patients deceased in a median time of 2.3 years (2.0 months—10.1 years; IQR 5.4 years) after their diagnosis of PPMF. Causes of death included lung cancer (*n* = 2), alcoholism (*n* = 2), liver failure, liver cirrhosis, multiple sclerosis, endocarditis, aspiration pneumonia, pulmonary embolism, trauma and intoxication. The cause of death in one patient was unknown; however, as this patient died 4.7 years after stent removal, it was considered unrelated to PPMF.

## Discussion

To date, our study represents the largest series of patients with PPMF secondary to pancreatitis who were initially treated endoscopically. Previous studies are predominantly case reports or review articles [14], the most extensive series being from India with 22 patients [10].

This study demonstrates the wide spectrum of symptoms of PPMFs, as well as their severity. Symptoms may range from mild abdominal pain or shortness of breath to severe sepsis requiring intensive care. Additionally, the time from onset to diagnosis varies. At times, symptoms lead to assessment for cardiac or pulmonary conditions, thereby delaying the diagnosis of PPMF.

In some patients, acute pancreatitis first occurred as long as 12 years prior to fistula formation. These findings highlight the importance of considering pancreas related diseases in patients with pleural effusion, especially combined with peripancreatic fluid collections, pancreatic calcifications or an earlier diagnosis of pancreatitis. Among our patients, pseudocyst or walled-off necrosis was seen in 75% and pancreatic ascites in 6.3% of cases. CT was an essential diagnostic tool in our study detecting the fistula in nearly all cases. MRI could provide an even more precise visualization of the fistula tract, although it is often unnecessary, as elevated pleural amylase as well as a CT-scan demonstrating chronic pancreatitis or pancreatic fluid collection are sufficient for a diagnosis of PPMF [15].

ERCP delineates the fistula tract as well and offers the advantage of simultaneous therapeutic intervention. In the present study, the fistula was identified by ERCP in 81% of cases which is slightly higher to the 70% reported by Kord Valeshabad et al. [16]. It may have been possible to visualize the fistula more often with increased volume of injected contrast but that would elevate the risk of PEP or infection of the fluid collection. In the treatment of disconnected ducts EUS-guided transmural drainage can be preferable option to ERCP [17].

A clear consensus on the management of PPMF has not been established. Conservative treatment is successful in only 16% of cases [18]. This study demonstrates that if the stent is positioned past the fistula tract, the success rate is high. However, when the area of disruption is in tail of the pancreas, this is usually not feasible. It is worth mentioning that among the five cases where ET was unsuccessful, there were four cases in which the fistula was in the tail of the pancreas. The remaining one case involved fistula located in the body of the pancreas. This patient underwent only two ERCPs and later deceased due to lung cancer.

It is not always possible to cannulate the PD, as happened in one case in our present series. Approximately 5% of patients with chronic pancreatitis have duodenal obstruction secondary to inflammation of the pancreatic head or groove pancreatitis, complicating transpapillary access [19]. As shown in the present study, when first PD cannulation fails, the second or third attempt may succeed. ET can obviate the need for surgical intervention. However, if it produces insufficient treatment results, it should not postpone inevitable surgery. Nonetheless, before operative treatment, it is of utmost importance to understand the anatomy of the pancreas. For instance, tail resection may result in a post-operative pancreatic leak from the resection site if there is an untreated obstruction in the distal part of the PD.

The risks of ET should also be considered. In our present study, the number of typical ERCP-complications was low. Stenting may cause a seeding of infection into a sterile pseudocyst or walled-off necrosis, as happened in one case. Assessment of infection in the fluid collection is complicated by the fact that PPMF can produce similar symptoms.

Previous studies have predominantly viewed PPMF as a disease affecting mainly middle-aged men [16]. In our study, this differed. As much as 42% of chronic pancreatitis and 33% of acute pancreatitis cases were female. Of the 19 female patients, 17 (89%) had alcohol-related pancreatitis. Therefore, PPMF should be considered in female patients with pleural fluid of unknown etiology. However, sex did not have a significant impact on treatment outcome.

Numerous comorbidities were reported among our patients, partly related to their unhealthy lifestyle. Heavy alcohol consumption and malnutrition hinder recovery. These factors, combined with our long follow-up period, help explain the high mortality rate of 27%.

A strength of this study is the large cohort of patients treated in a single center, where the treatment has been identical throughout the years – beginning with ET and proceeding to surgery if needed. With accumulated experience, our management of PPMF has evolved. For example, in the early years of this study, EUS was not available. In retrospect, some of the patients who underwent surgery could potentially have been treated with EUS. An additional strength of this study is the long follow-up period, with a median of 54 months. In contrast, follow-up in case reports is often limited to only a few days or months [20], thereby leaving possible recurrences unassessed. The retrospective nature is a limitation of this study. However, it was mitigated by excluding the patients with insufficient follow-up information.

## Conclusion

ET is an effective treatment method for PPMF. The primary goal of ET is to advance the PD stent beyond the fistula tract, as this is associated with treatment success. When the disruption is located in the pancreatic tail, the stent should be positioned as close as possible to the fistula tract. If cannulation of the PD is unsuccessful, EUS-guided transmural drainage should be considered. Should these treatment methods provide insufficient results, the pancreatic anatomy should be carefully evaluated prior to surgery. Future prospective studies of patients with IPF are needed to confirm the results of the present study.
